# Editorial: Rare diseases: from basic science to clinical practice and public health

**DOI:** 10.3389/fped.2025.1579709

**Published:** 2025-04-16

**Authors:** Urtė Oniūnaitė, Ozge Yilmaz, Rasa Ugenskiene, Lina Jankauskaite

**Affiliations:** ^1^ Department of Pediatrics, Lithuanian University of Health Sciences, Medical Academy, Kaunas, Lithuania; ^2^ Department of Pediatrics, Manisa Celal Bayar University, Manisa, Türkiye; ^3^ Department of Genetics and Molecular Medicine, Lithuanian University of Health Sciences, Medical Academy, Kaunas, Lithuania

**Keywords:** rare diseases (RD), paediatric, child, public health, quality of care, EURORDIS, RDCRN

**Editorial on the Research Topic**
Rare diseases: from basic science to clinical practice and public health

Rare diseases (RDs), characterized by their occurrence in a small proportion of the population, have garnered significant attention in recent years as a burgeoning public health concern (1). According to the Global Genes organization, more than 10,000 distinct rare and genetic diseases affect approximately 400 million individuals worldwide. Unfortunately, half of those diagnosed with RDs are children, and many of these conditions are either fatal or cause severe disabilities. Since RDs are heterogeneous, complex, and individually rare, diagnosing and assessing them poses substantial challenges, such as delayed diagnosis, inadequate support systems, persistent disability, and lack of effective treatment, often at substantial financial cost (1). It is estimated that up to 95% of rare diseases lack approved treatments by the United States Food and Drug Administration (FDA), with patients typically waiting an average of 4.8 years for an accurate diagnosis (2).

Accurate diagnosis and prompt treatment represent recurring hurdles for healthcare professionals working with RD patients. Advances in genetic testing and molecular biology have elucidated the pathogenesis of rare diseases and facilitated earlier diagnosis, leading to improved patient outcomes (3). Genetic testing expedites diagnosis and mitigates healthcare expenditures by circumventing unnecessary investigations, procedures, hospitalizations, and medication regimens, ultimately leading to the best possible clinical outcomes for patients (1). Innovations such as genetic modification and traditional drugs have provided breakthroughs against genetically determined diseases (4) while emerging research areas such as oligonucleotide therapy, stem cell therapy, and gene therapy have revolutionized treatment paradigms for myriad complex diseases (3).

The available options remain limited given the significant scientific expertise and investment capital required to develop treatments for rare diseases (3). Nevertheless, recent years have witnessed an outburst in mergers and acquisitions, driven by novel incentives and mitigation methods (5). Accelerated data analysis, integration, and aggregation of data facilitate the dissemination of information, the development of treatments, and new international collaborative research endeavors, thereby streamlining efforts for researchers, clinicians, patients, and their families (5). Standardization and streamlining of multi-site trial review and contracting processes are critical to improving patient access to new therapies, leveraging extensive datasets to foster novel medical insights, and refining trial designs (4). Thus, initiatives such as the Rare Diseases Clinical Research Network (RDCRN) or Rare Disease Europe (EURORDIS) encourage collaboration among diverse stakeholders, providing training opportunities for new investigators in rare disease research (3). Despite advances in technology, the 10-year survival rate post-diagnosis is still approximately 80%, with a significantly higher risk of death for men (6).

While developed countries have made significant advancements in addressing rare diseases, inequalities persist in less affluent regions, where access to healthcare improvements is limited, exacerbated by deficient public health infrastructure. Social customs and cultural practices rooted in history and tradition can hinder progress, particularly in regions with deep traditions (7). Varying definitions of rare diseases according to disease prevalence in different countries complicate intervention strategies.

Addressing rare diseases extends beyond pharmacotherapy to encompass holistic patient care, necessitating tailored interventions, symptom management, and environmental modifications to accommodate patient needs (7). The mental health toll exacted by RDs on affected children and their families is profound, resulting in psychological distress, diminished quality of life, heightened caregiver burden, and social upheaval (8, 9). The prolonged diagnostic journey, often culminating in unmet therapeutic needs, fosters prolonged uncertainty, fueling anxiety and emotional distress in patients and caregivers (8, 10). Even when patients are knowledgeable about their disease, knowing that the scientific understanding of the majority of rare diseases is lacking can increase anxiety about the future, create instability, and lead to various consequences even for family members (10). Additionally, caring for a sick child is highly time-consuming (due to hospital visits, specialized treatments, and diagnostic procedures since children require multidisciplinary care coordination across multiple sectors and a high frequency of inpatient stays), compounded by financial strain and disruptions to family dynamics that increase emotional distress (9). Siblings may feel overshadowed or neglected, giving up their free and leisure time. Moreover, there is a real medical financial hardship. All of this leads to higher emotional distress. Children afflicted with RDs encounter formidable barriers, such as compromised self-sufficiency and a lack of social support or understanding from others. They may also feel socially rejected and experience negative emotions from traumatic situations (e.g., separation from their parents during long hospital stays) (8, 9). Unfortunately, many families are unaware of psychosocial care services or do not have the time to seek them out, which makes psychological well-being even worse (8).

There is still a paucity of studies on the burden experienced by families of children with RDs. At present, the majority of family members report experiencing caregiver burden, which typically appears to increase in relation to the severity of the disease (9). Because RDs often start in childhood, the burden of the disease and its associated challenges most often fall on family members. Studies report that family members of individuals with RDs feel socially isolated and lonely (it is difficult for them to find other families facing the same disease and to share their caregiving burden); they also struggle to find enough time for themselves, which negatively impacts their work or occupation (9). It is worth mentioning that having a child with a rare disease places an emotional burden on family members and caregivers; there is a correlation between more debilitating forms of the disease and a greater impact on the family and their social life (9).

Children with RDs and their parents face multifaceted challenges, as existing healthcare models do not adequately address their medical and psychosocial needs. Communication barriers, compounded by speech and language disorders, impede daily care and social integration (10). Therefore, it is crucial to have good access to home healthcare services. However, due to funding problems and a need for more human resources, these families need more access to these services even in North America and Europe (10). Children with RDs and their families may encounter barriers to engagement in community activities due to a lack of accessible transportation and other adaptations to meet mobility or other needs. This creates significant barriers to attending medical appointments, engaging in educational and therapeutic activities, and playing, all of which require further adaptation to lead a full life.

In light of these challenges, several recommendations are warranted:

1. Active engagement of patients, families, and communities in the research process.
2. Adoption of a patient-centered model by healthcare companies leveraging that leverages digital and analytical technologies to personalize interventions.
3. To reduce the neglect and marginalization of the RD population, it is important to increase public awareness and competence in health and social care policies.
4. Comprehensive psychosocial assessments by healthcare providers to ensure appropriate support for patients and their family members.
5. Centralization of appropriate care, ensuring continuity, mitigating communication barriers, and enhancing access to specialist expertise, taking a broader view of the child's health and well-being than just one aspect of care by interacting with different medical recommendations from multiple specialists.

These measures collectively aim to optimize outcomes and improve the quality of life for individuals afflicted with RDs and their families.
